# Essential Experimental Methods for Identifying Bonghan Systems as a Basis for Korean Medicine: Focusing on Visual Materials from Original Papers and Modern Outcomes

**DOI:** 10.1155/2015/682735

**Published:** 2015-10-11

**Authors:** Hoon-Gi Kim, Byung-Cheon Lee, Ki-Bog Lee

**Affiliations:** ^1^Faculty of Liberal Education, Seoul National University, Room 208, Building 61, 599 Gwanangno, Gwanank-gu, Seoul 151-742, Republic of Korea; ^2^A324, KAIST Institute for Information Technology Convergence, Korea Advanced Institute of Science and Technology (KAIST), Yuseong-gu, Daejeon 305-701, Republic of Korea; ^3^Korea Atomic Energy Research Institute, 989-111 Daedeok-daero, Yuseong-gu, Daejeon 305-353, Republic of Korea

## Abstract

In the 1960s, through studies on Korean Medicine, Bonghan Kim proposed the Bonghan systems (BS) as the anatomical reality of the acupuncture meridians based on various experimental data. Since 2002, several groups, mainly led by a team at Seoul National University, who renamed the BS as the primo vascular system (PVS), have published around 70 papers showing biological structures corresponding to the BS. However, it is still difficult for other researchers to find them, especially under the skin, which Bonghan Kim first reported as acupuncture points, due to similar-looking biological tissues, for example, the lymphatic vessels, and such artifacts as blood clots or fascia debris. To solve these drawbacks, we examined the main methods for identifying the BS by comparing the original papers with the modern outcomes in terms of the common physical/chemical characteristics of the BS. In addition, effective methods of staining and microscopic observations discovered by modern teams are synthetically explained using visual materials such as diagrams and photos. Through the essentially organized methods in this review paper, we suggest that one can find the BS under the skin as putative acupuncture points by tracing the intraexternal BS, from which a new Korean Medicine will be born.

## 1. Introduction

Korean Medicine [1, 2] has developed in parallel with Chinese medicine. These two types of medicine have been established in the same root in which the meridian system has been considered as a vital channel holistically orchestrating the human body with the yin and yang. However, beyond the philosophical approach to the meridian system in China, in the 1960s a Korean named Bonghan Kim proposed the Bonghan system as an anatomical reality of the acupuncture meridian (Kyungrak in Korean) based on the various kinds of experimental data. A team led by Bonghan Kim at the Academy of Kyungrak, which was formerly called the Kyungrak Research Institute, published five research papers showing detailed anatomical/histological structures and biochemical components inside them for elucidating the new systems they found [3–7]. They called the structures Bonghan corpuscles (BCs) and Bonghan ducts (BDs) corresponding to the acupuncture points and meridian pathways, respectively. Although the team abruptly disappeared with their papers during the late 1960s in North Korea, and its claim was neglected in the academic field for about 40 years, Bonghan Kim remains the only scientist who has claimed to find the physical substances of the meridian systems thoroughly throughout the body using western scientific methodology.

Since 2002, however, a research group, called the Laboratory of Biomedical Physics for Korean Medicine in the Department of Physics, Seoul National University in South Korea, has taken the lead in representing the systems, publishing about 70 research papers in domestic and international journals [8]. In 2010, the team renamed the Bonghan system, the primo vascular system (PVS), and until recently, they reported many scientific evidences corresponding to the structures and contents of the BCs and BDs, as well as new findings regarding their distributions and functions that Bonghan Kim's research team (BRT) had not mentioned [9]. In addition, in the 2010s, other domestic and international teams, influenced by the PVS team, started to report the potential functions of the Bonghan system, which was related to tumor development [10], cancer metastasis [11, 12], and the origin of adult stem cells [13–15]. In 2013, the BRT's claim was academically reevaluated by international researchers, through the perspective of oriental medicine as well as western science, as a special issue titled “Primo Vascular System: Past, Present, and Future” with 18 articles in Evidence-Based Complementary and Alternative Medicine (http://www.hindawi.com/journals/ecam/si/954751/).

It is natural that recent researchers are mainly interested in the biological functions of the Bonghan systems. Particularly, the researchers of oriental medicine get to pay attention to the BRT's claim because it would imply the physical evidence of Qi circulation, which has been the basic premise of medical cures such as acupuncture and medicinal herbs for a long time.

However, for subsequent researchers, it is first necessary to determine the existence of the Bonghan systems themselves. Despite this necessity, there are some critical problems disturbing the formation of positive attitudes regarding the presence of the Bonghan systems. First, the BRT did not describe the methods used for identifying them, only revealing their locations within the body and their characteristics using experimental instruments such as several types of microscopes and dyes. For example, the BRT insisted that they first found the new anatomical tissues under some acupuncture points in the skin in the first paper without explaining experiment methods, and thereafter they showed them connected to the most organs in the second and third papers. Therefore, one can know whether the new structures are the real Bonghan systems after comparing the physical/chemical characteristics to those of the BRT papers, through numerous trial-and-error methods, such as in the works of the modern research teams (MRTs), particularly the PVS team. Second, even if one studies the methods identified from the papers of MRTs, there may still be difficulty in exactly identifying the Bonghan systems due to not only the existing biological tissues similar to them, but also the several kinds of artifacts brought about during the experiments. To find the Bonghan systems efficiently over such obstacles, it is necessary to have knowledge regarding the essential characteristics of the Bonghan systems through the papers by the BRT as well as the effective methods distinguishing them from artifacts suggested by the MRTs.

The purpose of this paper is to review the papers not only of the BRT but also of the MRTs to solve these problems. Comparing two kinds of papers, particularly using visual materials, the authors proposed the common essential characteristics about the Bonghan systems. In addition, the authors presented useful methods, through a summarizing of the MRTs' papers mainly with PVS team's ones, to avoid misjudgments from similar tissues and artifacts. This review paper may help subsequent researchers deeply study the Bonghan systems in the future.

## 2. Change from “Kyungrak” to “Bonghan Systems”

According to the second and third papers of the BRT, there are four kinds of Bonghan systems, that is, internal ones inside the blood and lymphatic vessels, intraexternal ones mainly on the surfaces of the internal organs, external ones in the skin (also independently named superficial systems) and through the outside vessels, and neural ones on the surfaces of the brain and inside the ventricles as well as in the central canal of the spinal cord, connected to the peripheral nervous systems. The BRT used the term “Kyungrak” for its newly discovered systems in the titles and texts throughout four of the papers, with the exception of the last one, which suggested the fundamental formation process for blood and white cells. Interestingly, however, the terms regarding the acupuncture points and meridian pathways in the text appeared almost only in the first paper, including sangyang (商陽) and igan (二間) on sooyangmyung daijanggyung (手陽明大腸經), joksamri (足三理) on jokyangmyung wikyung (足陽明胃經), rokoong (勞宮), haryum (下廉), soosamri (手三里), and hapkok (合谷) referring to* Dongeuibogam* (東醫寶鑑), which was written by a famous Korean medical doctor in 1610. Instead, the terms “Kyungrak” and “Bonghan systems” were used simultaneously in the second, third, and fourth papers without specific terms of the meridian systems, with the exception of joksamri, which was mentioned once in the second paper.

We guess that the change of naming was caused by the change in perception of the BRT through the new findings. In the first paper, the BRT reported that it tried to “clarify the material foundation of the basic theory” of oriental medicine and finally found the physical substance under the acupuncture points in the skin which were known to functionally be connected with internal organs such as the stomach and intestine. In the second paper, however, the BRT claimed that it observed a complex network throughout the whole body with a simple anatomical unit of BC and BD and the existence of the structure inside the blood and lymphatic vessels that “no one has ever conceived of.” To the BRT, these characteristics would clearly seem to be different from those of the meridian systems mentioned in traditional meridian literature.

Ironically, however, the MRTs were unable to find the external (and superficial) ones until recently. Therefore, in this paper, we investigated the three Bonghan systems and summarized the common characteristics reported by the BRT and MRTs. Also, we selected the first three papers [3–5] of the BRT for a comparison because the anatomical/histological structures as well as the biochemical components are mainly introduced in them. English versions, which were disclosed to the public online (http://www.ispvs.org/), were used because they contained colorful photos and diagrams unlike the Korean versions. For the papers of the MRT, the authors selected some critical photos clearly showing the characteristics of the Bonghan systems.

## 3. The Structures and Contents of the Bonghan Systems

### 3.1. The Basic Structures of the BD and BC Regarding Three Bonghan Systems

According to the BRT's first three papers, the basic unit of a Bonghan System consists of the BC and threadlike BDs. One BC connects with two or more BCs through the BDs, and all of them are semitransparent and look somewhat yellowish.

**Figure 1:**
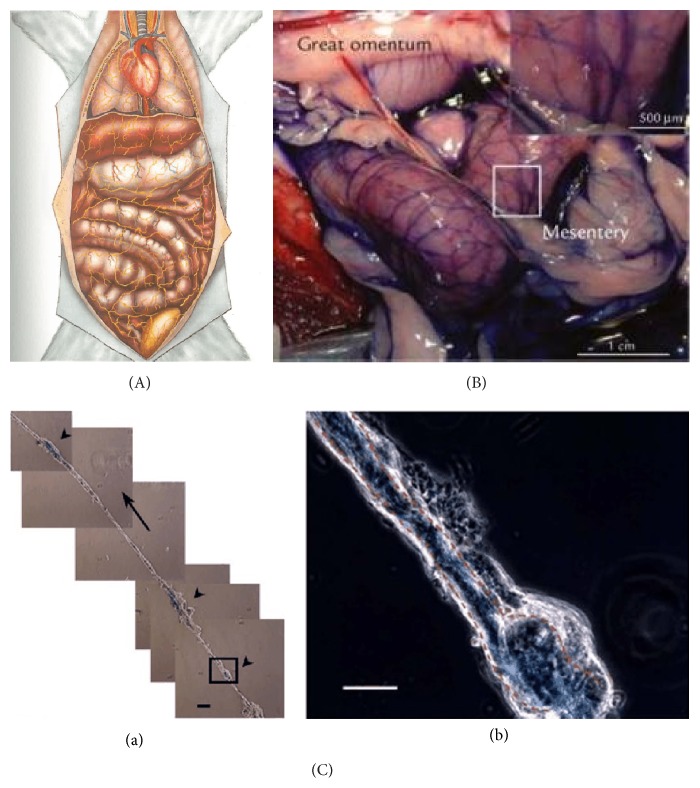
The intraexternal Bonghan systems. (A) The intraexternal Bonghan systems by the Bonghan research team. Diagram of the intraexternal Bonghan systems. The Bonghan ducts and corpuscles are distributed, independent of the paths of vessels and nerves, all throughout the body like a network. The Bonghan ducts exist in a free state, but they adhere to the surfaces of the organs and the walls of the blood vessels only in the region where they branch off [5]. (B) The intraexternal Bonghan systems stained by Trypan blue. Trypan blue-stained primo vascular system network on the mesentery of dog. The inset shows a high-magnified primo node (arrows) located at the joints of the primo vessels [16]. (C) The flow of liquor in the intraexternal Bonghan systems. The flow of Alcian blue (thick arrow, a) through a Bonghan duct connected to the corpuscles (arrowheads) on the surface of a rabbit's abdomen organ and a magnified view (b) of the boxed segment showing blue fluid inside the Bonghan duct and corpuscle (dotted red line) under a phase-contrast microscope. All scale bars are 50 *μ*m [17].

**Figure 2:**
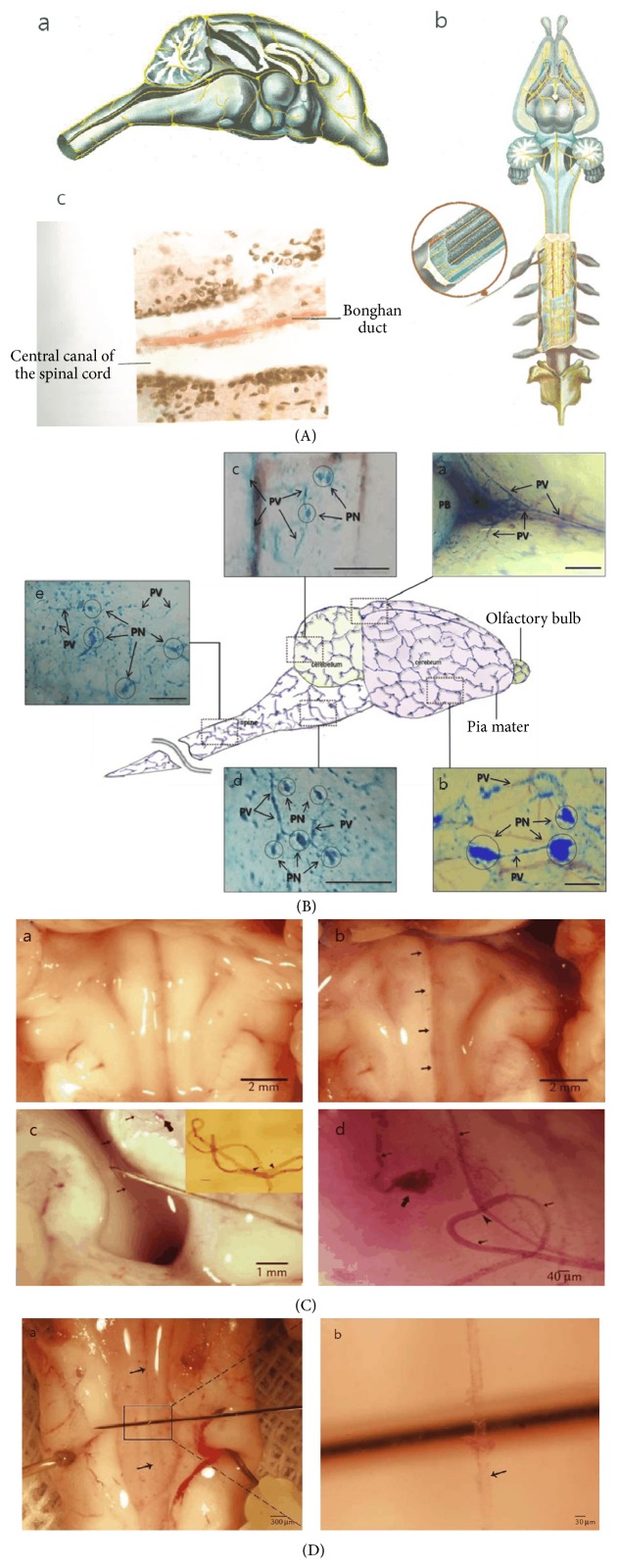
The neural Bonghan systems. (A) The neural Bonghan systems determined by the Bonghan research team. Diagrams and a photo of the neural Bonghan systems. In the central nervous systems, the Bonghan ducts (a, b) are distributed in the brain and spinal cord in a free state through the cerebral ventricles, the central canal, and the subarachnoid space along the circulating route of the cerebrospinal liquor. In addition, they enter under the perineurium and between the nerve fibers in the peripheral nervous systems. The Bonghan duct in the central canal of the spinal cord (c) is surrounded by unknown tissues [5]. (B) The Bonghan ducts and corpuscles on the surfaces of the brain stained by Alcian blue. Illustration of a network of the Bonghan ducts (PV) and corpuscles (PN) above the pia mater of the brain and the spine of rats stained by Alcian blue under a stereomicroscope. Stereoscopic images are visualized by spraying Alcian blue into the pia mater of the brain (a, b) and by injecting it into the lateral ventricles (c, d, e). The red-colored blood vessels are not stained (a, b, c). All scale bars are 500 *μ*m [18]. (C) The Bonghan duct in the brain ventricles stained by hematoxylin. The Bonghan duct in the brain ventricles of rabbits under a stereomicroscope. Image (a) at the bottom of the fourth ventricle beneath the cerebellum of a rabbit did not show any threadlike structures. However, after applying hematoxylin in the same region (b), the Bonghan duct (arrows) emerged near the sulcus. In addition, the Bonghan duct is stained by hematoxylin in the aqueduct and the third ventricle (c) and lifted using a needle to show that it was floating in the cerebrospinal fluid. The inset shows a wound state of the Bonghan duct specimen demonstrating its elastic nature and two Bonghan corpuscles (arrowheads). The scale bar of the inset is 60 *μ*m. Image (d) shows a Bonghan duct (arrows) with a corpuscle (thick arrow) and a node (arrowhead). One end of the structure was cut at the front part of the third ventricle [19]. (D) The Bonghan duct in the central canal of the spinal cord. The Bonghan duct (arrows, a) and its magnified view (b) inside an opened central canal of the spinal cord of a rabbit without dye treatment under a stereomicroscope [19].

**Figure 3:**
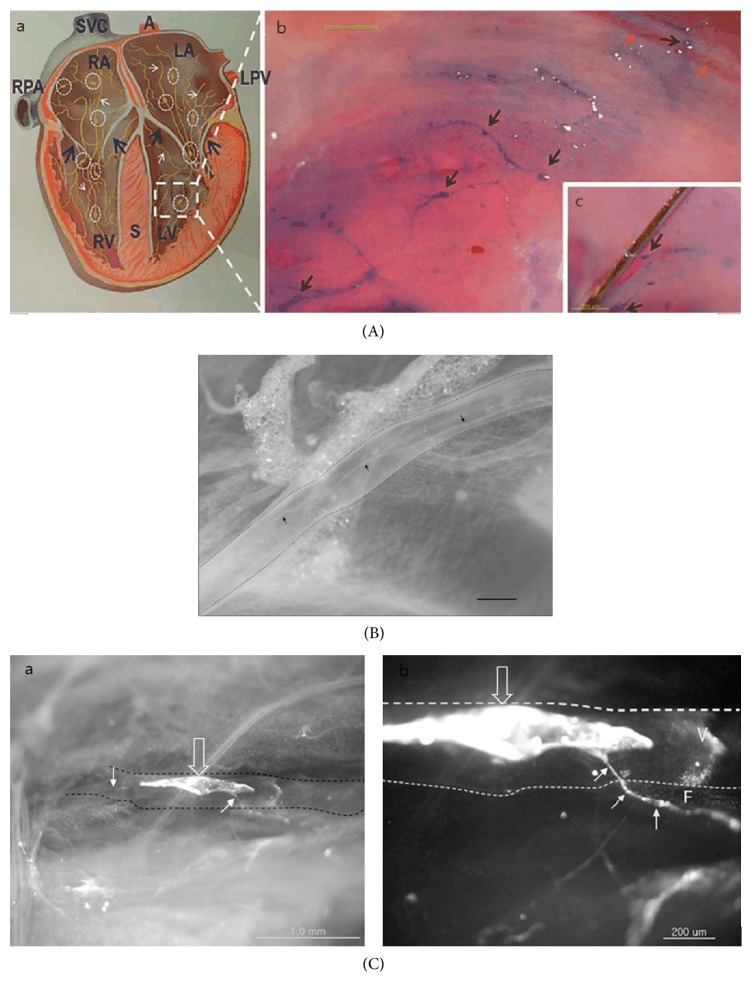
The internal Bonghan systems. (A) The Bonghan systems inside the heart stained by Trypan blue. Diagram from the third paper by the Bonghan research team (a), and photo images inside the bovine heart treating Trypan blue under a stereomicroscope by a modern research team (b and c). The Bonghan ducts (white arrows, a) and corpuscles (dotted circles) are indicated by the modern research team. SVC = superior vena cava; A = aorta; LA = left atrium; RA = right atrium; LPV = left pulmonary vein; RPA = right pulmonary artery; S = septum; LV = left ventricle; RV = right ventricle. The Bonghan corpuscles (black arrows, b) connected to the Bonghan ducts are stained blue, whereas the blood capillaries (red arrows, b) are not stained. The scale bar is 1 mm. The inset (c) demonstrates the lifting of a Bonghan duct using a needle showing the floating state of the Bonghan duct on the endocardium. The scale bar is 500 *μ*m [20]. (B) The Bonghan duct in the lymphatic vessel without staining. The Bonghan duct (arrows) in the lymphatic vessel (dotted line) on the caudal vena cava of a rabbit under a stereomicroscope using halogen red light. The scale bar is 500 *μ*m [21]. (C) The Bonghan duct in the lymphatic vessel stained by DiI. The Bonghan corpuscle (open arrow, a) and duct (arrows) in the lymphatic vessel (dotted line) on the caudal vena cava of a rabbit stained by DiI under a stereomicroscope. These are merges of bright-field and fluorescent images. A magnified view (b) shows that a Bonghan duct came out through the lymphatic vessel wall and entered the surrounding fat tissue (F). V is a valve weakly stained by DiI [21].

There are three major anatomical/histological characteristics of the BD. First, the number of BDs connected to one BC and their existing states differ depending on their locations in the body. Only two BDs are connected to one BC freely floating in the liquor, in case of those inside the blood and lymphatic vessels. However, in case of those on the surfaces of the abdominal organs, two or more BDs are reticulately connected to one BC spreading some branches into the organs (Figure 1(A)). For the neural Bonghan system, the BRT described the number of BDs as being only two to four. According to the visual materials, the spreading pattern inside the brain would be similar to the latter (Figure 2(A)(a-b)), and the pattern inside the central canal of the spinal cord would be analogous to the former (Figure 2(A)(b-c)). In addition, the pattern inside the heart, expressed only through a diagram, would be like the latter (Figure 3(A)(a)).

**Figure 4:**
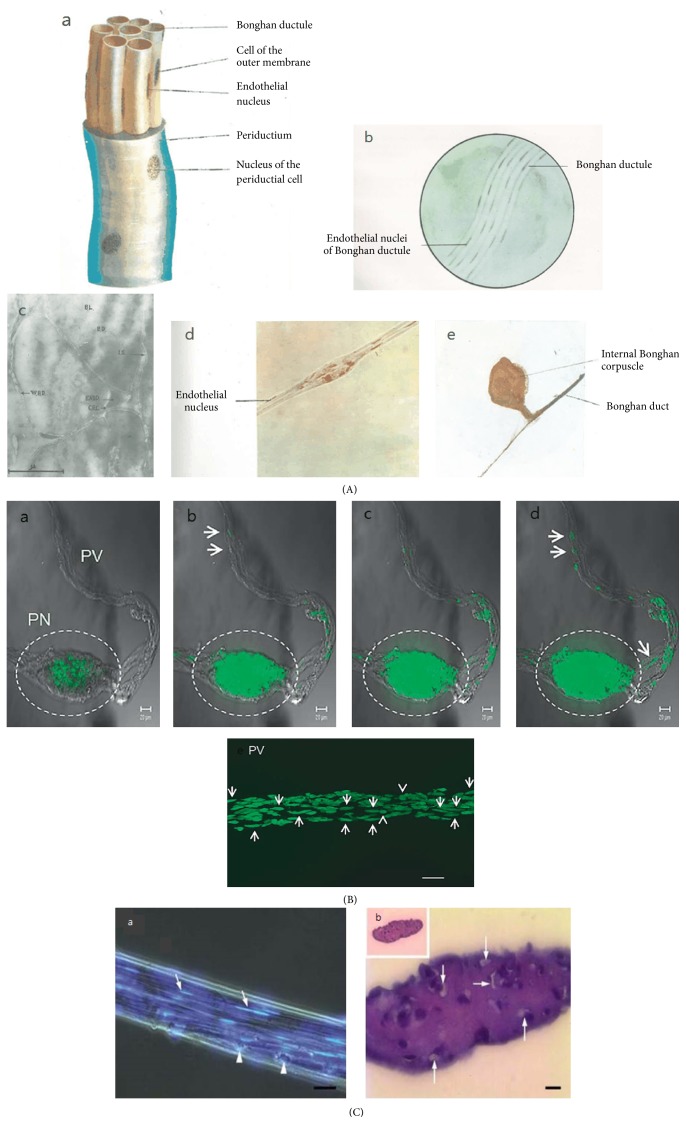
The structure of the Bonghan duct and corpuscle. (A) The structure of a Bonghan duct determined by the Bonghan research team. Diagram and photos showing the structure of the Bonghan duct and corpuscle. The Bonghan duct (a) consists of several subducts (Bonghan ductules) and three layers, and each layer has peculiar nuclei. Under a phase-contrast microscope, subducts and rod-shaped nuclei of endothelial cells (b) are observed. An electron micrograph of the internal BD (c) through a cross section (×42,000). BL = Bonghan liquor; BD = Bonghan ductule; IS = interstitial substance; WBD = wall of Bonghan ductule; ENBD = endothelial nucleus of Bonghan ductule; CEC = cytoplasm of endothelial cell. Photo image showing the rod-shaped nuclei of endothelial cells (d) from the intraexternal Bonghan duct stained using Feulgen reaction under a stereomicroscope (×400). Photo image (e) of the internal Bonghan duct and corpuscle (×63) [5]. (B) The Bonghan duct and corpuscle stained using acridine orange. The Bonghan duct (PV) and corpuscle (PV, dotted circle) inside the bovine heart stained by acridine orange under a confocal laser scanning microscope. The rod-shaped nuclei (arrows) are aligned longitudinally to the bundle of the Bonghan duct. Round nuclei (arrowheads, e) also exist. All scale bars are 20 *μ*m [20]. (C) The structure of the Bonghan duct. Histological observation of the Bonghan duct, whole specimen and cross-sectioned. Photomicrograph (a) of merged phase-contrast and fluorescent image of a Bonghan duct, which shows the bundle of several ductules (arrows) with characteristic rod-shaped nuclei stained with DAPI (bright blue) and immune cells (arrowheads) on duct surface. The scale bar is 50 *μ*m. Cross-sectioned Bonghan duct (b) also presents several ductules (arrows). The scale bar is 10 *μ*m [22].

Second, a BD consists of a bundle of ductules (Figure 4(A)(a–d)). Each ductule has two layers, an endothelial layer and an outer membrane. These are totally surrounded by the other layer, the periductium. Generally, the diameter of a ductule reaches 5–15 *μ*m, but it sometimes varies by 1–50 *μ*m (Table 1). The BRT also showed a cross section of the internal BD under an electron microscope (Figure 4(A)(c)). The authors believe that the bundle structure is important to distinguish from other biological tissues and systems because the blood and lymphatic vessels consist of only one duct, and the nervous system and connective tissues are not a duct system.

Third, the three layers mainly consist of some kinds of cells, which have peculiar types of nuclei, respectively. Among them, the nuclei of the endothelial cells, 15–20 *μ*m in length, take a rod shape with pointed ends and are more intensively stained than the others (Figure 4(A)(a-b, d)). These nuclei align longitudinally along the ductule. The authors guess that the shape of the nuclei and the type of arrangement within the endothelial layer is another distinct point between the BD and existing circulatory vessels. The endothelial cells of the blood and lymphatic vessels have somewhat rod-shaped nuclei but with blunt ends, and the nuclei align vertically as well as longitudinally through the vessels.

Meanwhile, the BC's form is oval or spindle-like (Figure 4(A)(e)) and is surrounded by one layer. Generally, the layer of biological tissues can consist of cells, fibers made of proteins, or a mixed form of them. The BRT, however, did not indicate the exact ingredients about the BC layer, just mentioning that there is a thin membrane made of connective tissues containing oval or spindle-like nuclei of cells for the neural BC in the third paper.

The Bonghan ductules inside the BC become enlarged and form sinuses, which become ramified and tangled. Around the sinuses, there are undifferentiated reticular tissues such as those of hemopoietic organs. The size of the BCs varies depending on their locations in the body (Table 1).

### 3.2. The Contents of the BD and BC in Three Bonghan Systems

According to BRT's first three papers, one of the most abundant elements inside the ductules is nucleic acid, that is, DNA and RNA. They are categorized by three types by the size: basophile granules, basophile structures of various shapes, and nucleus-like structures. Among them, in the fourth paper, spherical basophile granules are called “Sanals,” meaning “live eggs” in Korean, whose center consists of abundant DNA surrounded by RNA, ranging from 1.2 to 1.5 *μ*m in diameter. The BRT confirmed the presence of nucleic acids in the granules and structures inside not only the ductules but also the BC, by a Feulgen reaction and Brachet reaction (or Unna-Pappenheim reaction), which has been available for identifying DNA and RNA in a nucleus, respectively. In addition, the BRT used acridine orange for identifying the DNA elements inside the superficial Bonghan systems in the second paper (in the fourth paper, the BRT produced the content and base composition of DNA and RNA in the Sanals through a quantitative analysis). Another interesting discovery within the ductules is the presence of chromaffin granules, which appeared to be a positive response of adrenalin reaction by Hillarp's method.

In the BCs, there are granules and structures consisting of nucleic acids and chromaffin granules. However, certain kinds of cells are observed in the BCs unlike in the ductules. One of them is the chromaffin cell, which has plenty of chromaffin granules inside. The existence of chromaffin granules and cells suggests that the Bonghan systems contain adrenomedullary hormones such as adrenalin and noradrenalin. In the third paper, the BRT reported that there are more adrenalin and noradrenalin than any other biological tissues and organs as a result of analyzing the liquor components of the Bonghan systems. Moreover, an adrenal cortical hormone like corticosteroid and a sex hormone like estrogen are found there. Another kind of cell, just observed in the internal BCs, is the family of hematopoietic cells such as myelopoietic and lymphogenetic cells in different stages of differentiation, that is, granulopoietic, monopoietic, erythrogenic, and lymphopoietic elements and megakaryocytes. Moreover, only in the internal BCs, there is a group of cells similar to the peculiar parenchymal cells of the organs. The types of the cells depend on the positions of the internal BCs. For example, in the internal BCs of the liver, there are cells similar to the liver parenchymal cells.

According to the third paper, hyaluronic acid, a kind of mucopolysaccharides, is another rich component inside the Bonghan systems. In general, it has been known that hyaluronic acid significantly contributes to cell proliferation and migration and distributes throughout connective, epithelial, and neural tissues. The BRT mentioned that there are more hyaluronic acids in the Bonghan systems compared to other biological tissues and organs but did not explain how they had come to notice this. The other materials contained in the Bonghan systems are biogenic elements for cell metabolism such as proteins, lipids, carbohydrates, free amino acids, and mononucleotides.

## 4. How to Distinguish the Bonghan Systems from the Surrounding Tissues

In general, Bonghan systems could be obtained from live animals. The BRT anaesthetized animals such as rabbits, rats, and mice and found these systems under living conditions. The authors conjecture that it may be very difficult to posthumously identify the Bonghan systems because they rapidly deteriorate through the postmortal changes. For example, the BDs inside the blood and lymphatic vessels may aggregate among themselves or stick on the vessels while the liquor flow in the vessels gradually stops. However, the sampling inside the heart inevitably needs to proceed after slaughter. In this case, the state of the heart should be prepared as freshly as possible [20]. In the case of the sampling for the brain Bonghan systems, it is generally possible to obtain them after decapitation, but the sample could be obtained after piercing holes and injecting some dyes into the head of an anaesthetized animal for minimizing the biological change brought by decapitation [23].

Although the BRT mentioned the sizes and shapes of the BDs and BCs, it would be hard to discriminate them from the surrounding tissues such as lymphatic capillaries and lymphoid nodules with similar sizes and semitransparency like threads and corpuscles. In fact, it has been pointed out for a long time in the academic fields that the MRTs as well as the BRT had misjudged the lymphatic systems as new structures. In addition, the fascia, thin membrane surrounding all organs in the body, is another biological tissue that makes it difficult to find the Bonghan systems, because the BDs and BCs are inserted into the semitransparent fascia exposing only some parts of them outside. For instance, the peritoneum, which is one of the serous membranes, consists of three layers: superficial fascia, deep fascia, and subserous fascia. In the case of the central nervous systems, the dura mater, arachnoid mater, and pia mater protect the brain and the spinal cord. The Bonghan systems are distributed through the interior and the surface of these kinds of fascia, as suggested in a MRT's model [24, 25]. Sometimes, the mesentery, a fold of the anterior and posterior walls of the peritoneum, looks like a threadlike structure under the naked eye or a stereomicroscopic view.

The BRT indicated that the anatomical/histological structures of the Bonghan systems are different from those of the lymphatic systems and the fascia but did not inform how to exactly recognize them. Therefore, the MRTs had to find the Bonghan systems developing its own specialized microscopic instruments and dyes.

One of the methods to distinguish Bonghan systems from the lymphatic systems would be to observe the former inside the latter simultaneously* in situ* through a microscope without any dyes. One MRT found a way to do so in the lymphatic vessel of rabbits (Figure 3(B)), using a stereomicroscope with a red light from a halogen light source [21]. Without that light, the lymphatic vessel under a stereomicroscope was observed to be almost transparent, containing nothing inside. This method cannot be applied for a blood vessel because of its red constituents (Table 2).

In addition, the MRTs developed some dyes selectively staining the Bonghan systems not only in the lymphatic vessels but also in the fascia of the organs. A prime example is Trypan blue, which has been useful to identify the Bonghan systems on the surfaces of the organs such as the heart (Figure 3(A)), the intestine (Figure 1(B)), and the brain [16, 20, 23] under a stereomicroscope or a light microscope. Through the experimental processes, the MRTs concluded that Trypan blue could preferentially stain the Bonghan systems rather than the lymphatic vessels and the fascia as well as the blood vessels, nerves, and adipose tissues. However, the MRTs did not explain what the mechanism of staining was in the reports. Nevertheless, the fact that Trypan blue has been available for preferentially staining the Bonghan systems is interesting because it has been used to separate live cells from dead cells by staining the proteins of the latter in the biological fields. In general, Trypan blue has been known to not pass through the membranes of viable cells.

In addition, one MRT found that a dye, hematoxylin (Figure 2(C)), which forms salts with basophilic compounds such as DNA and RNA, could preferentially stain the BD in the brains of rabbits [19]. The BRT had used hematoxylin for staining the nuclei of endothelial layer's cells of the ductules in the third paper. The idea of the MRT came from the concept that the nucleic acids inside the Bonghan systems can also be discriminatively stained by hematoxylin. In the same experiment, the MRT was sometimes able to find the BD in the central canal of the spinal cord without hematoxylin, which was connected along the BD from the ventricles of the brain (Figure 2(D)).

The MRT developed another dye, 1,1′-dioctadecyl-3,3,3′,3′-tetramethylindocarbocyanine perchlorate (DiI), to selectively reveal the membranes of the Bonghan systems. DiI is a fluorescent lipophilic dye and has often been used for tracing the nervous systems as they have lots of lipophilic components. The MRT conceived that DiI (Figure 3(C)) could stain the phospholipids of the membranes and successfully presented the Bonghan systems in the lymphatic vessels of rabbit [21] as well as rat [26] and brain [19] under a stereomicroscope. Some images stained by DiI were particularly notable because they showed that the BDs enter into the nearby tissues or organs by passing through the lymphatic wall.

Moreover, the MRTs paid attention to the mentions of the BRT in which there is more hyaluronic acid than any other biological tissues in the fluid inside the BDs. The MRTs discovered that Alcian blue, a dye used for staining some types of acidic mucopolysaccharides, could make the fluid of BD blue and clearly showed the stained BDs (Figure 2(B)) on the surface of the brain [18] and in the lymphatic vessels [21, 27] under a stereomicroscope by injection or spraying.

## 5. How to Distinguish the Bonghan Systems from the Potential Artifacts

### 5.1. Identifying Process in the Sampling Stage

The most common artifact in the sampling stage is made from blood clots. When the blood vessels are damaged during surgical processes, the biological self-defense mechanism forming thrombus immediately is activated in the body. Fibrins, threadlike proteins polymerized from fibrinogens, form a network trapping the blood cells. Therefore, it is difficult to distinguish the Bonghan systems from blood clots. This situation frequently comes along in finding Bonghan systems in the blood vessel systems including the heart. Thus, it would be practically reliable to get them in the relatively thick blood vessels such as the caudal vena cava, iliac vein, and thoracic aorta. The thrombus, however, forms right after incising the blood vessels during a surgery to find the Bonghan systems. Moreover, blood clots can be generated in the lymphatic vessels. For example, the threadlike structures removed from the lymphatic vessels could be a mixture of fibrins in the lymph with lymphocytes. However, it should be noted that the Bonghan systems would exist while mixed with the thrombus.

Another typical artifact, particularly on the surface area of the visceral organs, is the threadlike structures generated from the fascia during dissection. Generally, the fascia contains not only cells such as fibroblasts and fibrocytes, but also fibers made of proteins such as collagen fibers, reticular fibers, and elastic fibers. If one incises the fascia to find the Bonghan systems on the surfaces of the organs, they can usually observe a threadlike mass around the incision sites due to the aggregation of cells and fibers.

**Figure 5:**
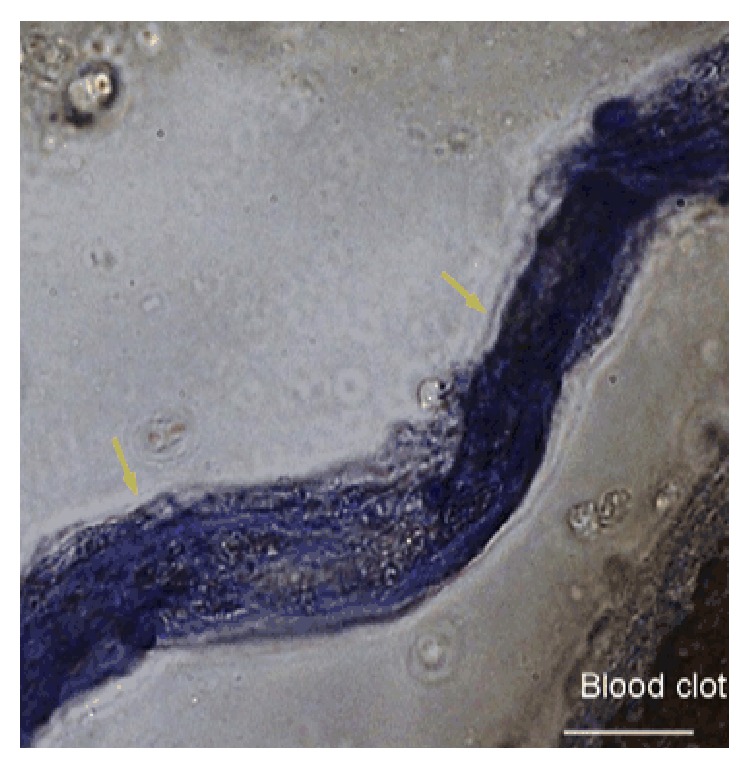
The Bonghan duct and blood clot stained using Cr-Hx. The Bonghan duct (arrows) in the venous sinuses of a rat brain protruded from a blood clot is stained blue by Cr-Hx under a light microscope. Cr-Hx nearly does not stain the blood clot. The scale bar is 12 *μ*m [28].

Beyond those artifacts, are there any reliable methods to identify the Bonghan systems? One MRT thought that the Bonghan systems can be preferentially stained by some dyes rather than the artifacts, which have no nuclei. One of the dyes discovered by the MRT was chromium-hematoxylin (Cr-Hx), which strongly stains chromaffin granules or cells as well as acid materials such as nucleic acids. The MRT found that Cr-Hx could preferentially stain the Bonghan systems compared to blood clots (Figure 5) under a light microscope [28]. The authors think that treating Cr-Hx is available in the experiment sites not only for identifying the Bonghan systems in blood clots, but also for ignoring something with no staining such as blood clots without Bonghan systems and the fascia fibers made of proteins. In addition, as mentioned above, Trypan blue and DiI are useful for distinguishing Bonghan systems from the fascia.

### 5.2. Confirming the Presence of the Ductules and Rod-Shaped Nuclei

Although one can observe the Bonghan systems using some specific microscopes and staining suggested by the MRTs, the possibility of causing artifacts still remains in the process of obtaining “pure” systems. One of the reasons for this is that it is difficult to adjust the proper incubation time of the dyes. When the incubation time for staining is too long, nearby biological tissues can be contaminated in addition to the Bonghan systems. In this case, one could get the wrong tissues, for example, the threadlike and corpuscle structures made by fibers and fibroblasts of the fascia. In addition, it is possible that the chemical reactions between some dyes and existing vessels could bring about unsuspected byproducts. For example, uncertain threadlike structures can be formed owing to the chemical reactions between the dyes and inner walls of the blood or lymphatic vessels.

To isolate the pure Bonghan systems over such artifacts, it is necessary to first confirm whether the threadlike structures of the sample have ductules containing rod-shaped nuclei. The BRT showed several visual images of the ductules containing the rod-shaped nuclei with pointed ends of endothelial cells through the diagrams, the photos under a phase-contrast microscope, and the photos stained by some dyes under a stereomicroscope (Figure 4(A)(a–d)). The BRT mainly used a Feulgen reaction and hematoxylin to stain the nuclei of endothelial cells.

The MRTs also showed rod-shaped nuclei with pointed ends within the bundle by an advanced instrument, a confocal laser scanning microscope (CLSM), presenting high-resolution images from selected depths. The MRTs observed the Bonghan systems using staining methods such as a Feulgen reaction [29], acridine orange [20, 21, 30], and Janus Green B [31] treatment. The photo images (Figures 4(B) and 4(C)) show that the BD is different from the blood or lymphatic vessels because there are ductules forming a bundle and the rod-shaped nuclei inside aligned longitudinally through the ductules. The existence of rod-shaped nuclei also implies that this structure is not a thrombus formed from the aggregation of fibrins or fascia fibers with blood cells, not only because there is no nucleus in the red blood cells and platelets, but also because the shapes of the white blood cells are round or irregular. On the other hand, the MRTs presented the lumens or sinuses inside the BD [17, 18, 20, 22, 31] under several kinds of electron microscopes such as a scanning electron microscope (SEM), cryo-SEM, focused-ion-beam-SEM, and transmission electron microscope (TEM). In addition to the above criteria for confirming real primo vessels, it would be much more reliable to consider that primo vessels should be distinguished from inflammatory substance as Wang et al. suggested [32].

### 5.3. Confirming the Presence of the DNA and Chromaffin Substances inside the Bonghan Systems

**Figure 6:**
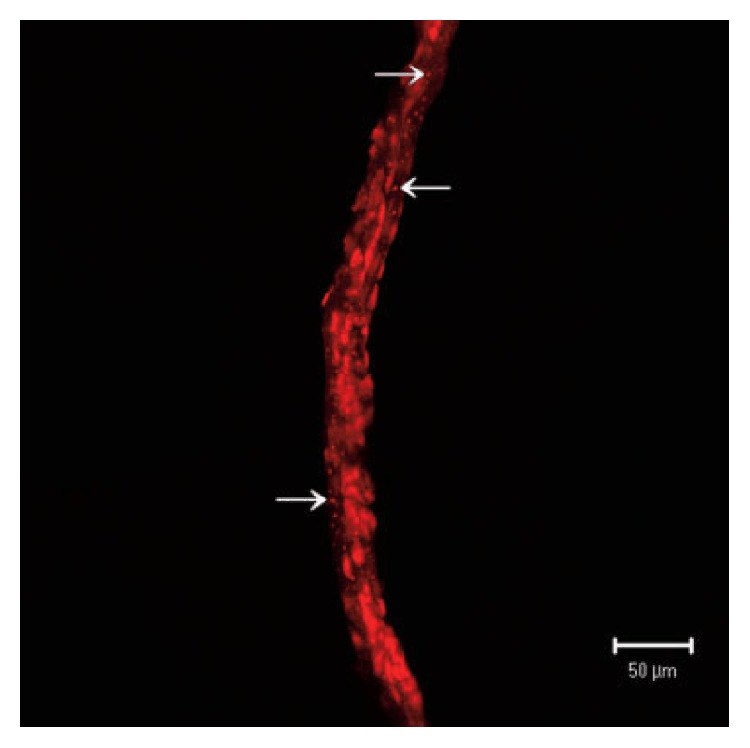
The DNA substances inside the Bonghan duct stained through a Feulgen reaction. The 1-2 *μ*m granules (arrows) revealing the DNA content inside the subducts of a Bonghan duct at a rabbit's abdomen surface, stained using a Feulgen reaction under a stereomicroscope [29].

**Figure 7:**
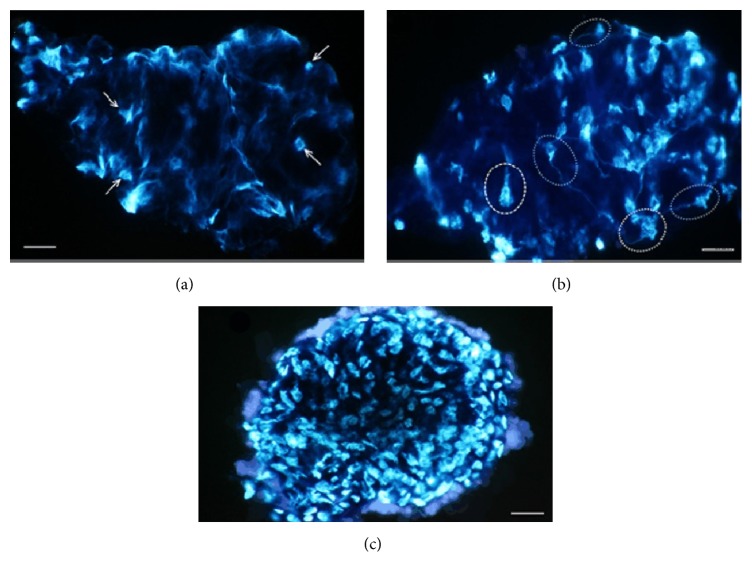
The dynamics of DNA substances inside a Bonghan corpuscle stained using DAPI. Three kinds of extracellular DNA (eDNA) in the BC above the pia mater of a rat brain and spine, stained using DAPI under a stereomicroscope. The BC has eDNA with rare nucleus-like forms (arrows, a). Most eDNA images appear amorphous. The BC has many nucleus-like forms (dotted circles, b) connected to threadlike structures. The eDNA images show normal nuclei (c). The scale bars are 25 *μ*m, 28 *μ*m, and 29 *μ*m, respectively [18].

The MRTs have found some types of DNA components in the Bonghan systems using a qualitative analysis. For example, one MRT noticed granules of 1-2 *μ*m in size in the subducts in the BD of the abdomen surfaces of rabbits (Figure 6) using a Feulgen reaction [29]. In addition, inside the BC of the rats' brain and spine, another MRT [18] observed three types of extracellular DNA (eDNA) stained by 4′,6-diamidino-2-phenylindole (DAPI), a fluorescent dye binding DNA, under a fluorescence microscope (Figure 7). This observation implies that the DNA substance inside the BC can transform into nucleus-like structures.

The existence of DNA components, inside the Bonghan systems as well as in the endothelial cells of the ductules, means that the staining methods for nuclei can distinguish Reissner's fibers, the glycoprotein fibrous structures from the subcommissural organ through inside the ventricles and central canal of the spinal cord, from the Bonghan systems. Because they are found in the central nervous systems of most vertebrates, their main function not being well known, they could be confused with the Bonghan systems during the experiments. They mainly consist of carbohydrates and proteins without cells. The MRT showed that the structures they had found were well stained with dyes for nuclei, such as DAPI and yoyo-1, and confirmed that they were not Reissner's fibers. In addition, another MRT observed that the structures were well stained by DiI. Because Reissner's fibers have no cell membranes, they are not stained by DiI [19].

The presence of chromaffin substance would be proved because one MRT clearly stained the BD and BC by Cr-Hx [28], which stains the chromaffin granules or cells, as mentioned above. In addition, the MRTs reported the presence of cells of a mixed type with adrenalin and noradrenalin [33] or chromaffin cells [34] on the surface of the mammals' abdomen organs in the BCs.

## 6. Conclusions

Although the BRT claimed that it found the anatomical/histological structures corresponding to acupuncture points and meridian pathways referring to the knowledge of Korean Medicine [1, 2], it is still unclear whether BCs and BDs are really the parts of meridian systems or not [35]. In fact, the BRT showed only a few examples for meridian systems associated with the structures in the skin it found in the first paper. Besides, the BRT reported that the distribution of the structures coincides with acupuncture points indicated in* Dongeuibogam* “as a whole,” not concretely. The BRT even said that there are some more acupuncture points not reported before.

If the MRTs including the researchers on Korean Medicine succeeded in finding the BCs which the BRT reported in the skin, the relationship between the Bonghan systems and the meridian systems would be clearly determined. However, until recently, the Bonghan systems in the skin have not reported in the academic fields.

We guess that one of the ways to find the superficial BCs is the backtracking method. For example, one could trace the structures connected from the intraexternal Bonghan system to near the skin using some dyes or radioisotopes. To do this, the basic step is to identify Bonghan systems correctly without any artifacts in the experimental process. We also think that the relationship itself between the traditional acupuncture points and the BCs is not the critical problem for Korean Medicine. Instead, the idea of the close relationship among four Bonghan systems in the body is more meaningful to the fields of diagnosis and cure for Korean Medicine [1, 36], which has almost the same concept with the BRT's.

Although the BRT described in detail the characteristics of the Bonghan systems through its first three papers, it has not been repeatedly possible to find them in animals. The main reason seems that the Bonghan systems usually exist within the existing biological tissues or organs, that is, inside the blood vessels, the lymphatic vessels, the heart, the ventricles, and the central canal of the spinal cord. Some parts of them appear on the surface of the organs such as the intestines and brains, but many of them exist in the fascia. No one has been able to easily find these new structures, which have not been reported in the academic fields. Another reason is the possibility of artifacts during the surgery process. One can be confronted with several kinds of artifacts caused by physical damage or chemical reaction with the dyes, from the artifacts themselves such as blood clots to the mixed forms of the Bonghan systems, with artifacts such as the BDs surrounded by erythrocytes or lymphocytes in the vascular and nervous systems. Even the BDs connected to the corpuscle-like blood clots can be found. To escape from such difficulties, the authors tried to present the main methods for identifying the Bonghan systems more easily in this document, comparing the works of the MRTs to those of the BRT with the visual materials.

As for the three Bonghan systems, the authors have confirmed that the MRTs can find most of the basic anatomical/histological structures and inner biochemical components corresponding to the research results of the BRT. However, until recently, many things reported by the BRT can be found only in part or not at all. For instance, the Bonghan systems in the peripheral nervous systems and the RNA substance inside the BD or BC have not been reported. In addition, the MRTs found some kinds of cells, which partly resembled the cells from the BRT paper inside the internal Bonghan systems, such as immune cells and hematopoietic cells through a TEM observation or immunohistochemical analysis [13, 14].

In addition, the point most stressed by the BRT is the function of the DNA granules, Sanals, which grow into new cells and are generated from the existing cells flowing through the entire body. The BRT insisted that the circulation of the liquor containing Sanals be confirmed through the dosimetry of radioactivity and microradioautography [4–7]. Although one MRT [17] partly showed that the liquor in the BD flows in one direction (Figure 1(C)), a presentation regarding the circulation of Sanals and liquor still remains an important problem to be proved. To examine the structures and functions of the Bonghan systems more deeply, we suggest that the first step for exactly identifying the Bonghan systems free from artifacts at the experimental sites is critical. In addition, we recommend that, for data consistency, one should use and show the same samples taken from the Bonghan systems through all of the processes.

## Figures and Tables

**Table 1:** The size of a Bonghan corpuscle and ductule determined by the Bonghan research team.

	Bonghan corpuscle			Bonghan ductule
	Internal	Intraexternal	Neural	
Size	0.1–0.2 mm	Connecting to two Bonghan ducts 0.3–1.0 × 0.1–0.5 mm	0.5–1.0 × 0.2–0.5 mm	Generally 5–15 *μ*m
		Connecting to three or over Bonghan ducts 0.6–2.5 × 0.3–1.5 mm		Totally 1–50 *μ*m

**Table 2:** The experimental procedure for identifying and confirming the Bonghan systems.

Stage	Target	Checkpoint	Experimental methods	
			Stain	Microscopy
Identifying the BS				
From the other tissues	Inside the lymphatic vessel	—	—	Stereomicroscope with a red light
	On the surfaces of the organs	Preferentially stained	Trypan blue	Stereomicroscope, light microscope
	In the brain	Preferentially stained	Hematoxylin	Stereomicroscope
	Inside the lymphatic vessel and in the brain	Preferentially stained (especially from Reissner's fibers)	DiI	Stereomicroscope
	Inside the lymphatic vessel and on the brain	Inner fluid stained	Alcian blue	Stereomicroscope
From the artifacts	In the blood clots and the fascia	Preferentially stained	Chromium-hematoxylin	Stereomicroscope, light microscope

Confirming the BS	In the Bonghan duct	Ductules in a duct containing the rod-shaped nuclei of endothelial cells	Feulgen, acridine orange, and Janus Green B	Stereomicroscope, CLSM, SEM, and TEM
	In the Bonghan ductule	DNA stained	Feulgen, DAPI, and yoyo-1	Stereomicroscope, fluorescent microscope
	In the BS	Chromaffin substance stained	Chromium-hematoxylin	Light microscope
